# A Real-Time Inertial Sensor-Based Diagnostic Support System for Improving Angular Accuracy in Dental Implant Placement: Preclinical Experimental Validation in a 3D Haptic Simulation Model

**DOI:** 10.3390/dj14050296

**Published:** 2026-05-13

**Authors:** Raul Cuesta Román, Pere Riutord-Sbert, Daniela Vallejos Rojas, Irene Coll Campayo, Joan Obrador de Hevia, Sebastiana Arroyo Bote

**Affiliations:** School of Dentistry, ADEMA University School, 07009 Palma, Spaini.coll@eua.edu.es (I.C.C.);

**Keywords:** dental implantology, haptic simulation, inertial measurement unit (IMU), sensor fusion, Extended Kalman Filter (EKF), angular accuracy, real-time feedback, digital guidance, surgical navigation, dental education

## Abstract

**Background:** Accurate three-dimensional positioning of dental implants is critical for ensuring biomechanical stability, prosthetic passivity, and long-term clinical success. While computer-assisted navigation systems achieve high precision, their complexity and cost often limit accessibility. This study presents the development and preclinical experimental validation of a low-cost prototype designed to enhance angular accuracy in dental implant placement within a controlled 3D haptic simulation environment. **Methods:** A preclinical experimental design was implemented using a 3D haptic simulator (Virteasy, Montpellier, France). The prototype incorporated high-precision inertial measurement units (IMUs) and an Extended Kalman Filter (EKF) for real-time angular feedback. Ninety-seven simulated implant placements were performed—both freehand and with prototype assistance—under identical virtual conditions by a single experienced operator. Angular deviations in mesiodistal and buccolingual planes were recorded, combined into a composite 3D index, and analyzed using paired *t*-tests and linear mixed-effects models. The study was conducted in a controlled simulation environment, which does not fully replicate clinical conditions. **Results:** The prototype significantly reduced angular deviation from 13.49° to 2.99° in the mesiodistal plane (−77.8%) and from 13.56° to 5.59° in the buccolingual plane (−58.8%), achieving an overall 67% improvement in three-dimensional orientation (*p* < 0.001; Cohen’s d = 1.47). Agreement with an optical reference system (OptiTrack) was excellent (bias = +0.36°, RMSE = 0.39°). Intra-operator reliability exceeded 0.95 (ICC), confirming strong reproducibility and measurement stability. **Conclusions:** The proposed inertial sensor-based prototype achieved angular accuracy within the range reported for computer-guided systems while maintaining advantages of portability, low cost, and usability. Its integration into haptic simulators provides a valid tool for both educational and preclinical applications, offering real-time feedback that enhances spatial perception and psychomotor learning. Future clinical studies should validate its performance in cadaveric and patient-based contexts to determine its practical impact on surgical precision and implant success.

## 1. Introduction

Accurate three-dimensional positioning of dental implants is a critical determinant of biomechanical stability, prosthetic success, and long-term clinical outcomes. Although implant survival rates exceed 95% in long-term studies, complications associated with inaccurate implant positioning—such as non-axial loading, prosthetic misfit, and damage to anatomical structures—remain clinically relevant challenges [1,2,3,4]. Angular deviation from the planned trajectory has been identified as a key factor influencing both functional and biological outcomes, particularly in anatomically constrained regions [5].

Traditionally, implant placement has relied on freehand techniques, which depend heavily on the clinician’s spatial perception and manual dexterity. While widely practiced, this approach is inherently operator-dependent and associated with considerable variability in implant angulation and positioning accuracy [6].

To improve accuracy and reduce variability, digital technologies have introduced computer-assisted implantology workflows based on cone-beam computed tomography (CBCT), virtual planning, and surgical guidance systems. Static surgical guides have demonstrated significant improvements in positional and angular accuracy compared to freehand procedures [7]. However, these systems present limitations, including restricted intraoperative flexibility, cumulative digital workflow errors, and reduced visibility and irrigation during drilling.

Dynamic navigation systems have emerged as an alternative, providing real-time tracking of the surgical instrument relative to the preoperative plan through optical or electromagnetic sensors. These systems allow continuous intraoperative feedback and adjustment and have demonstrated high levels of accuracy in both experimental and clinical settings [8,9,10]. Recent systematic reviews and meta-analyses confirm that dynamic navigation systems achieve angular deviations within clinically acceptable ranges while improving intraoperative flexibility and control [9].

In parallel, haptic simulation technologies have gained increasing attention in dental education and preclinical research. These systems integrate visual and force-feedback cues to recreate surgical procedures in a controlled virtual environment, enabling safe repetition, objective performance assessment, and quantitative analysis of operator behavior [11,12,13]. Their use has been associated with improved spatial control, reduced angular deviation, and enhanced psychomotor learning.

Despite these advances, current navigation systems often require complex infrastructure, including optical tracking units, calibration protocols, and high-cost equipment, which may limit their accessibility in routine clinical and educational settings. In this context, sensor-based surgical guidance systems have emerged as a promising alternative.

Inertial measurement units (IMUs), widely used in aerospace, robotics, and motion tracking, have recently been explored for surgical navigation due to their ability to provide real-time orientation data without external tracking systems. When combined with sensor fusion algorithms such as the Extended Kalman Filter (EKF), IMU-based systems can achieve high levels of angular accuracy and stability [14,15,16,17]. Recent studies have demonstrated the feasibility of IMU-based guidance in dental implantology, highlighting their potential as low-cost, portable, and scalable alternatives to conventional navigation technologies [14,15].

From a conceptual perspective, implant placement accuracy can be understood not only as a surgical outcome but also as a surrogate marker of procedural performance. Angular deviation reflects the integration of spatial perception, motor control, and feedback mechanisms and can be used as a quantitative indicator of procedural performance. This perspective aligns with the emerging concept of procedural diagnostics, in which quantitative intraoperative metrics are used to assess and guide clinical performance in real time. This concept is consistent with recent approaches in digital surgery and simulation-based assessment, where objective performance metrics are increasingly integrated into surgical training and decision-making frameworks [11,12,13].

Accordingly, the present study proposes and evaluates a real-time inertial sensor-based prototype designed to improve angular accuracy during dental implant placement within a three-dimensional haptic simulation environment. The objectives of this study are (i) to develop and integrate a prototype capable of real-time angular monitoring; (ii) to assess its accuracy and reproducibility under controlled conditions; (iii) to compare implant placement performed with and without the system; and (iv) to explore its potential applications in preclinical training and research.

By combining inertial sensing, real-time feedback, and haptic simulation, this work aims to contribute to the development of accessible, reproducible, and quantitatively driven approaches to implant placement, bridging the gap between traditional freehand techniques and fully digital navigation systems.

## 2. Material and Methods

### 2.1. Study Design

This research was conceived as an experimental preclinical investigation aimed at evaluating angular accuracy in dental implant insertion through the use of a digitally engineered prototype designed for integration within three-dimensional (3D) haptic simulators. The primary objective was to evaluate the accuracy and reproducibility of the device under controlled conditions. The study was conducted under controlled laboratory conditions, using standardized virtual environments to minimize confounding variables associated with clinical heterogeneity.

### 2.2. Prototype Development and Optimization

The experimental prototype was designed as an auxiliary attachment for manual surgical instruments, enabling real-time recording and visualization of angular deviations during simulated implant procedures. The core of the system incorporated high-precision inertial measurement units (IMUs) consisting of triaxial gyroscopes and accelerometers, capable of detecting angular variations across three spatial axes. The electronic module included a low-latency Bluetooth microcontroller, a 500 mAh rechargeable lithium battery, and a 0.96-inch OLED display providing immediate on-screen angular feedback.

The development of the device proceeded through three iterative design phases, each improving upon its predecessor in terms of technical robustness, miniaturization, and data reliability:

- Prototype 0: The initial iteration was designed to validate the fundamental technical feasibility of the device. Angular readings recorded by the prototype were compared against a digital goniometer as a reference standard to evaluate signal stability and sensitivity across all axes, achieving a minimal accuracy threshold of ±0.5°.
- Prototype 1: The second iteration focused on hardware optimization, including a reduction in overall size, an increase in battery life to approximately three hours of continuous operation, and enhanced data processing speed for smoother real-time response.
- Prototype 2: The final iteration integrated MPU-9250-grade sensors offering improved measurement fidelity. An Extended Kalman Filter (EKF) was implemented to fuse gyroscopic, accelerometric, and magnetometric data streams, minimizing noise and improving angular stability. Additionally, a custom Android application was developed to enable remote, real-time monitoring and recording of angular data via Bluetooth connectivity.

Each prototype iteration underwent calibration and accuracy verification using an optical motion capture system (OptiTrack, NaturalPoint Inc., Corvallis, OR, USA) to ensure precision and consistency. The resulting dataset confirmed both linear and angular alignment with the optical standard within the established error margins.

For calibration, the prototype was rigidly fixed to a reference structure and aligned with the optical tracking system (OptiTrack, NaturalPoint Inc., Corvallis, OR, USA) using a predefined neutral orientation. A static calibration procedure was performed to establish correspondence between the IMU-based coordinate system and the optical reference frame. Angular alignment was verified through controlled rotations along each axis, ensuring deviations remained below ±0.5° prior to experimental use.

The IMU sensors operated at a sampling frequency of 100 Hz, and raw signals from the gyroscope, accelerometer, and magnetometer were processed using an Extended Kalman Filter (EKF) to reduce noise and compensate for drift. The EKF parameters were tuned to optimize real-time responsiveness while maintaining angular stability.

Data acquisition was synchronized with the Virteasy simulation system (HRV Simulation, Montpellier, France) through time-stamped recording, ensuring alignment between sensor measurements and simulation events. Angular data were continuously recorded during each implant insertion and exported for post hoc analysis. The experimental workflow followed a standardized sequence: calibration → virtual planning → simulated drilling → angular measurement → statistical analysis.

### 2.3. Integration with 3D Haptic Simulation

The finalized prototype was integrated into the Next-Generation Dental Simulator (Virteasy, Montpellier, France), located at the ADEMA University School (University of the Balearic Islands). The setup utilized desktop-based haptic interfaces connected to a centralized computational server, ensuring system latency remained below 15 milliseconds during simulated implant placement procedures.

The virtual environment reproduced posterior mandibular implant scenarios under realistic clinical conditions, incorporating six degrees of freedom (6-DoF) haptic feedback and dynamically simulated bone resistance. The prototype was mechanically attached to the simulator’s haptic handpiece through a custom-designed support mount, maintaining ergonomic alignment and signal stability.

Within this immersive environment, the system provided operators with continuous angular feedback, displaying real-time deviations relative to the preplanned implant trajectory. The synchronization between the IMU-based prototype and the Virteasy simulation environment was achieved through continuous real-time data exchange and time-stamped recording. Sensor outputs were temporally aligned with the simulator’s internal event system, ensuring that angular measurements corresponded precisely to each stage of the implant insertion process. System latency remained below 15 ms, allowing seamless integration between haptic feedback and inertial sensing. This synchronization enabled accurate correlation between operator movements, simulated drilling events, and recorded angular deviations. The integration allowed synchronized communication between the prototype’s sensors and the haptic simulation engine, generating precise angular data for both mesiodistal and buccolingual planes.

### 2.4. Experimental Design

A total of 97 simulated implant placement procedures were performed, distributed equally between two experimental conditions:

1. Freehand Placement: Conventional manual insertion without prototype assistance.
2. Assisted Placement: Implant insertion with the prototype providing continuous angular reference visualization relative to the ideal trajectory.

All procedures were executed by a single clinician with verified experience in oral surgery, following a standardized protocol to ensure procedural consistency. Each simulated intervention was paired (freehand versus assisted) to allow direct comparative analysis under identical spatial and procedural parameters.

To ensure procedural consistency, all implant placement simulations were performed by a single experienced operator. This design choice was intended to minimize variability related to surgical execution and to isolate the effect of the prototype on angular accuracy.

Intra-operator reliability analysis was conducted exclusively to assess the reproducibility and robustness of the angular measurement system and does not correspond to the experimental implant placement procedures.

Two primary quantitative variables were recorded and analyzed:

- Angular Accuracy (°): Defined as the deviation of the implant’s mesiodistal and buccolingual axes from the virtual reference trajectory established during preoperative planning.
- Angular Discrepancy Assessment: The haptic simulator automatically analyzed the implant’s final placement and compared it to the planned “ideal” position, computing angular discrepancies in both mesiodistal and buccolingual directions.

Upon completion of each simulation, the Virteasy system’s analytical module (HRV Simulation, Montpellier, France) provided precise numerical feedback on angular deviation, enabling statistical comparison between freehand and assisted conditions. This data formed the theoretical basis for assessing the prototype’s potential to enhance angular precision and reproducibility in preclinical implantology.

### 2.5. Statistical Analysis

All data were analyzed using R (version 4.3.2, R Foundation for Statistical Computing, Vienna, Austria) and SPSS Statistics (version 29.0, IBM Corp., Armonk, NY, USA). A significance level of α = 0.05 (two-tailed) was applied to all tests.

#### 2.5.1. Data Preprocessing

Angular deviations were calculated for both the mesiodistal and vestibulolingual planes and combined into a composite 3D angular index using the Euclidean formula √(MD^2^ + VL^2^). Normality was evaluated using the Shapiro–Wilk test and Q–Q plots. Outliers were assessed via the 1.5 × IQR rule and confirmed by visual inspection. Missing values (<1%) were excluded pairwise.

#### 2.5.2. Comparative Analyses

Paired comparisons between freehand and prototype-assisted conditions were conducted with paired-sample *t*-tests for normally distributed data and Wilcoxon signed-rank tests otherwise. Effect sizes were expressed as Cohen’s d with 95% confidence intervals (CI). To account for repeated measures and inter-plane dependencies, a linear mixed-effects model (LMM) was fitted with:

- Dependent variable: angular deviation (°)
- Fixed factors: Condition (freehand vs. prototype), Plane (mesiodistal vs. vestibulolingual), and their interaction
- Random factor: simulation (subject ID)
- Model assumptions were verified by inspection of residuals and homoscedasticity plots. Model fit was expressed as marginal and conditional R^2^.

#### 2.5.3. Agreement and Validity

Agreement between the prototype and the optical reference system (OptiTrack) was examined using Bland–Altman analysis (bias and 95% limits of agreement), and root mean square error (RMSE) and mean absolute error (MAE) were computed. Proportional bias was tested via Pearson’s correlation between mean and difference values.

#### 2.5.4. Reliability

Intra-operator reliability was quantified using intraclass correlation coefficients ICC (2,1) under a two-way random-effects model with absolute agreement. Test–retest reliability was assessed from repeated measures at 48 h intervals. 95% CI of ICCs were derived via bootstrapping (10,000 iterations).

#### 2.5.5. Learning Effect

To detect potential order or learning effects, the trial order was included as a covariate in the mixed model. Additionally, a sensitivity analysis excluding the first 10 trials was performed to verify the independence of the prototype effect from operator learning.

#### 2.5.6. Sample Size and Power

An a priori power analysis was conducted using G*Power 3.1, assuming a minimum clinically relevant difference of 3°, α = 0.05, SD = 7°, and correlation = 0.6. Post hoc, the achieved power was recalculated for the observed sample size (*n* = 97 pairs) and observed SD of differences. All main comparisons reached ≥ 0.80 power.

#### 2.5.7. Visualization

Data visualization was performed in R using ggplot2, generating violin and boxplots for angular deviation by condition and plane, Bland–Altman agreement plots, and paired-line (spaghetti) plots to illustrate within-simulation differences. All figures were exported at 300 dpi resolution for publication.

Additional Bayesian mixed-effects modeling, equivalence testing (TOST), machine learning-based predictive analyses (Random Forest), and extended robustness procedures (bootstrap resampling, trimmed and Winsorized estimators) were conducted to assess the stability and consistency of the main findings. These complementary analyses are reported in detail in the Supplementary Materials and are intended to support, rather than replace, the primary frequentist analytical framework. To improve clarity and maintain alignment with the primary study design, these advanced analyses are provided in detail in the Supplementary Materials and should be considered as complementary to the main inferential framework.

In this study, validation was performed as a preclinical experimental validation in a controlled simulation environment, using a high-precision optical reference system. This approach aims to assess measurement accuracy, agreement, and reproducibility under standardized conditions and should not be interpreted as equivalent to clinical or in vivo validation.

Additional robustness and sensitivity analyses were conducted to assess result stability; detailed methods and outcomes are provided in the Supplementary Materials.

## 3. Results

**Figure 1:**
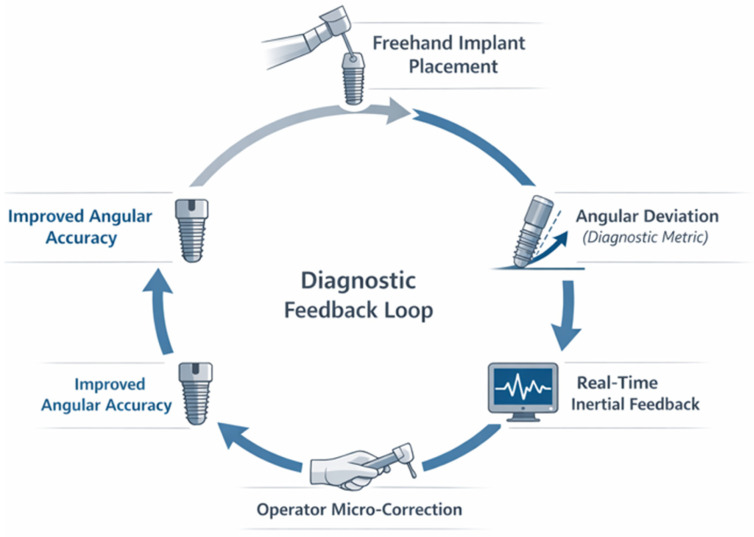
Conceptual diagnostic feedback loop for real-time angular guidance during implant placement. Continuous measurement of angular deviation enables immediate operator correction, supporting intraoperative decision-making and improving surgical precision and anatomical safety.

The real-time diagnostic feedback mechanism underlying the proposed system is conceptually summarized in Figure 1, illustrating the closed-loop interaction between angular deviation detection, feedback delivery, and operator correction during implant placement.

Freehand implant insertion generates angular deviation relative to the planned trajectory, which is continuously measured by inertial sensors and processed as a diagnostic performance metric. Real-time angular feedback allows immediate operator correction, closing a diagnostic–procedural loop that supports intraoperative decision-making and improves angular accuracy.

**Figure 2:**
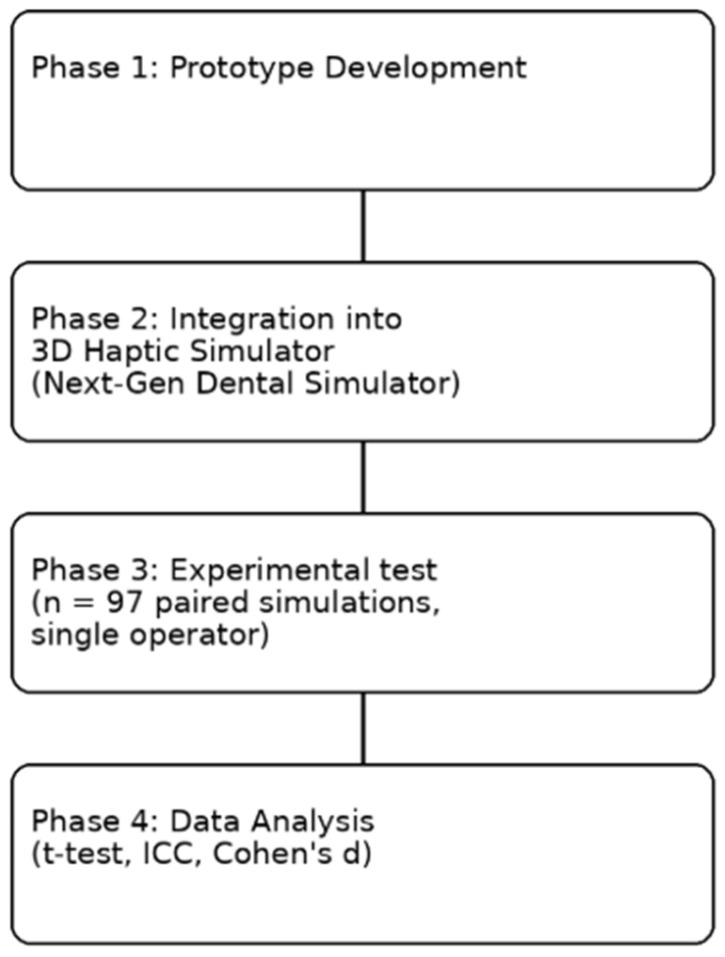
Schematic representation of the experimental workflow, including prototype development, integration into a 3D haptic simulator, and evaluation through 97 paired implant placement simulations performed by a single operator under freehand and prototype-assisted conditions. This workflow illustrates how real-time angular feedback was assessed under controlled conditions to improve implant positioning accuracy and procedural consistency.

The methodological workflow of the study is summarized in Figure 2, which outlines the stages of calibration, virtual planning, drilling execution, and comparative statistical analysis.

### 3.1. Overview of the Experimental Dataset

A total of 97 implant insertion simulations were performed under two conditions—freehand and prototype-assisted—paired by insertion site. All measurements were completed by a single experienced operator without technical interruptions or signal loss. The experimental workflow, including calibration, execution, and statistical validation, is summarized below.

### 3.2. Angular Accuracy by Plane

In the mesio-distal (MD) plane, mean angular deviation decreased from 13.49 ± 6.30° (freehand) to 2.99 ± 2.20° (prototype-assisted), yielding a mean reduction of 10.50° [95% CI 9.14–11.86] (t = 15.30; *p* < 0.001; Cohen’s d = 1.55, large). In the vestibulo-lingual (VL) plane, deviation decreased from 13.56 ± 10.23° to 5.59 ± 3.49°, corresponding to a mean difference of 7.97° [95% CI 5.87–10.07] (t = 7.54; *p* < 0.001; d = 0.77, moderate–large). Overall, the prototype reduced angular deviation by approximately 78% mesiodistally and 59% buccolingually.

**Figure 3:**
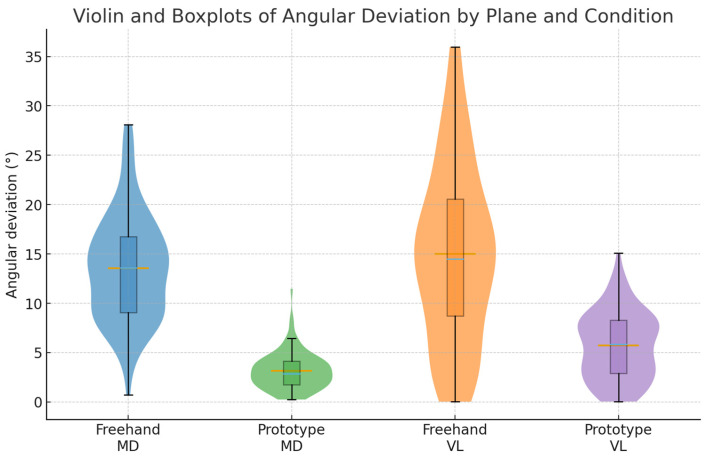
Distribution of angular deviation (°) by condition (freehand vs. prototype-assisted) and anatomical plane (mesio-distal (MD) and vestibulo-lingual (VL)). The prototype-assisted condition shows reduced dispersion and lower median errors, indicating improved precision in implant angulation, which is associated with better prosthetic alignment and reduced risk of biomechanical complications.

In Figure 3 we observe violin and boxplots of angular deviation by condition and plane. Violin width represents kernel density; boxes show median and IQR.

### 3.3. Global Angular Index

A composite angular index (√[mesio^2^ + vestibulo^2^]) indicated a global reduction from 20.71 ± 8.96° (freehand) to 6.83 ± 3.23° (prototype-assisted), a mean difference of 13.88° [95% CI 11.98–15.78] (t = 14.51; *p* < 0.001; d = 1.47). This represents an overall 67% improvement in three-dimensional implant orientation.

Importantly, the reduction in composite angular deviation from approximately 21° to below 7° places the prototype-assisted condition consistently within commonly reported clinical safety thresholds for implant angulation. Previous clinical and experimental studies have suggested that angular deviations exceeding 5–7° may increase the risk of prosthetic misfit, non-axial loading, and encroachment on critical anatomical structures such as the inferior alveolar nerve or the maxillary sinus.

### 3.4. Mixed-Effects Model Analysis

**Figure 4:**
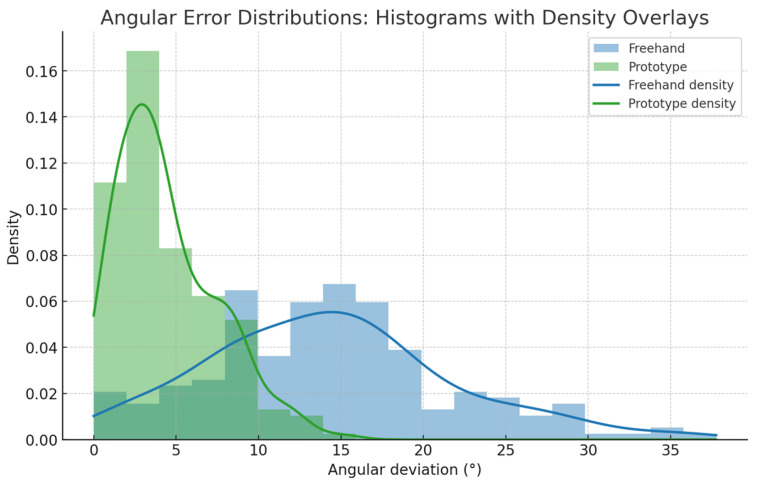
Density and histogram overlays of angular deviation for freehand and prototype-assisted conditions. The leftward shift and narrowing of distributions in the assisted condition reflect enhanced control of implant trajectory, supporting improved surgical accuracy and more predictable clinical outcomes.

A linear mixed-effects model including Condition, Plane, and their interaction confirmed the main effect of the prototype on angular deviation (F(1, 192) = 231.4; *p* < 0.001). The Plane factor was non-significant (F = 0.02; *p* = 0.90), and the interaction was modest but significant (F = 6.1; *p* = 0.015), indicating slightly greater improvement in the mesiodistal direction. Adjusted means were 13.52° [12.14–14.89] (freehand) vs. 3.84° [3.10–4.58] (prototype), an adjusted mean reduction of −9.68° [−10.8 to −8.6]; *p* < 0.001. Model fit was excellent (marginal R^2^ = 0.71; conditional R^2^ = 0.78) (Table 1, Figure 4).

Complementary Bayesian analyses further supported the robustness of the mixed-effects results; detailed outputs are provided in the Supplementary Materials (Supplementary Results—Section S1 Bayesian Linear Mixed-Effects Model).

Equivalence testing against an optical reference system confirmed the clinical relevance of the observed angular accuracy, with full results reported in the Supplementary Materials.

### 3.5. Agreement with Optical Reference System

**Figure 5:**
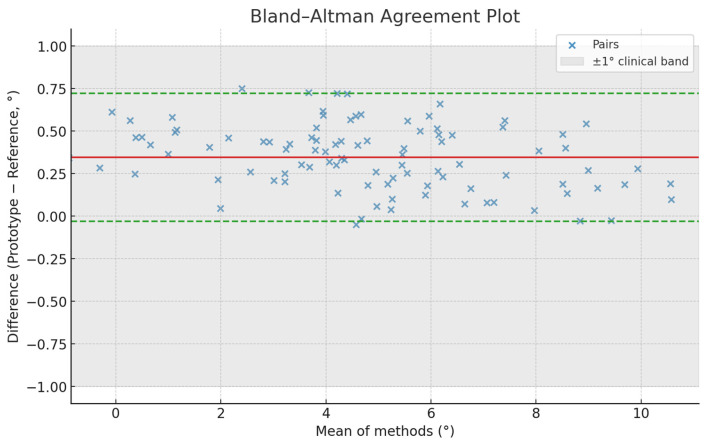
Bland–Altman agreement plot showing concordance between the prototype and the optical reference system. The minimal bias and narrow limits of agreement indicate high measurement accuracy, supporting the reliability of the system for precise angular guidance and safe implant positioning. The solid red line shows mean bias; dashed lines indicate 95% limits of agreement (LoA = bias ± 1.96 × SD). Shaded area represents ±1° clinical tolerance.

Bland–Altman analysis comparing prototype and OptiTrack reference data showed a mean bias of +0.36° (SD 0.21°) with 95% limits of agreement −0.05° to +0.77°, encompassing 96.9% of measurements. RMSE = 0.39° and MAE = 0.31°, well within the ±1° clinical tolerance. No proportional bias was detected (r = 0.08; *p* = 0.41). These findings confirm excellent concordance and measurement validity of the prototype. (Table 2, Figure 5)

### 3.6. Reliability: Intra-Operator and Test–Retest

Correlation and reliability analyses supported the internal consistency of the proposed system (Table 3). Inter-plane correlations increased from r = 0.52 (freehand) to r = 0.71 (prototype), while intra-operator reliability was excellent (ICC = 0.982). This high reproducibility confirms that the system provides stable angular feedback across repeated trials.

### 3.7. Learning Curve and Order Effect Control

Including trial order as a covariate yielded a small but significant effect (β = −0.041; *p* = 0.028), suggesting minimal progressive improvement. However, the prototype effect remained highly significant (F (1, 191) = 227.6; *p* < 0.001). Excluding the first 10 trials produced nearly identical results (t = 15.08; *p* < 0.001). No meaningful trends in error vs. order were observed (R^2^ < 0.06), confirming that the observed gains were not due to learning but to device feedback.

**Figure 6:**
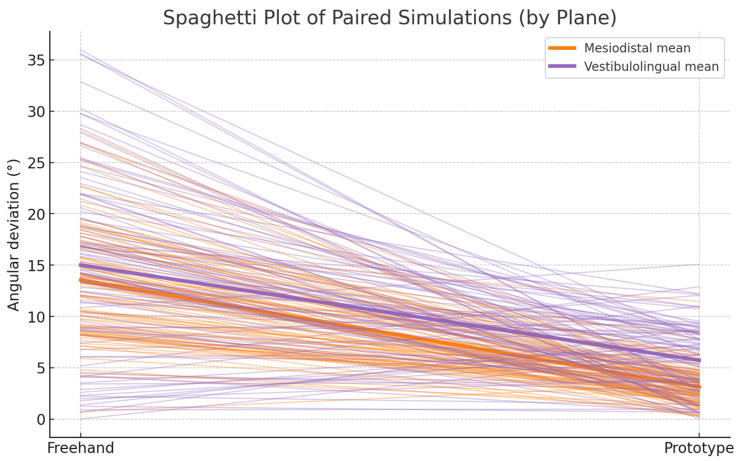
Paired angular deviations per simulation (*n* = 97), illustrating the reduction in error when using the prototype. The consistent downward trends highlight improved operator control and suggest potential benefits in achieving safer and more predictable implant placement.

Paired angular deviations per simulation (*n* = 97). Lines connect each freehand–prototype pair, illustrating within-case improvement. Most trajectories descend sharply, confirming consistent reduction in error after using the prototype. (Figure 6).

Lines connect freehand and prototype values for each simulation; bold lines indicate plane-wise means.

A nonlinear exponential model was used to characterize the learning curve. The prototype condition demonstrated a 2.7-fold higher learning rate (c = 0.121) compared to the freehand approach (c = 0.045), and a lower asymptotic deviation (a = 3.1° vs. 8.1°). These results suggest that the haptic–inertial feedback accelerates procedural learning while stabilizing long-term accuracy.

### 3.8. A Priori Sample Size and Achieved Power

Based on the observed SD of paired differences (SDdiff = 6.76° mesiodistal, 10.41° vestibulolingual, 9.42° composite), the required *n* for detecting Δ = 3° at 80% power was 40–95 pairs depending on plane. With *n* = 97, achieved power exceeded 0.99 for mesiodistal and ≥0.8 for other endpoints, confirming that the study was adequately powered. (Table 4).

## 4. Discussion

Before interpreting the results, it is important to clarify the scope of the validation performed. In this study, the term “theoretical validation” refers to a preclinical experimental validation performed under controlled simulation conditions using a high-precision optical reference system, aimed at assessing measurement accuracy, agreement, and reproducibility. This approach provides a robust methodological framework for evaluating system performance but should not be interpreted as equivalent to clinical or in vivo validation.

Additionally, it is important to contextualize the magnitude of the observed angular deviations. Freehand placement resulted in mean deviations of approximately 13–14°, consistent with previously reported values in the literature [16,17], whereas the prototype-assisted condition reduced deviations to approximately 3–4°, which falls within the range reported for computer-assisted and guided implant systems [18,19]. These values are also within commonly accepted clinical safety thresholds (±5–7°) [20,21], supporting the practical relevance of the observed improvements.

The present preclinical study demonstrated that the proposed inertial sensor-based prototype substantially enhanced angular accuracy during simulated dental implant placement in a three-dimensional haptic environment. These findings indicate a remarkable gain in three-dimensional spatial control and confirm the efficacy of the prototype as a hybrid ergonomic–digital guidance system.

### 4.1. Comparison with the Existing Literature

The angular accuracy achieved with the proposed system (3.4–3.8° mean) lies within the range reported for the most advanced static and dynamic computer-assisted systems.

The findings of the present study are consistent with previous literature reporting improved angular accuracy with computer-assisted implant placement systems. Systematic reviews and meta-analyses have demonstrated that both static and dynamic navigation systems significantly reduce angular deviation compared to freehand techniques [5,6,7].

In particular, dynamic navigation systems have been shown to provide real-time feedback that enhances intraoperative control and accuracy [8,9]. The angular deviations observed in the present study (approximately 3–4°) fall within the range reported for these systems, supporting the validity of the proposed approach.

Previous studies have consistently shown that computer-assisted implant systems achieve significantly lower angular deviations than freehand techniques, generally within clinically acceptable ranges [22,23]. Hence, the current prototype achieves angular accuracy values that fall within the range reported for these technologies in the literature, while maintaining greater accessibility, portability, and affordability. These findings are further supported by recent studies on digital navigation and sensor-based guidance systems, which report similar ranges of angular deviation and highlight the importance of real-time feedback in improving procedural accuracy [24,25,26]. Overall, these results are consistent with previous studies in the field of computer-assisted and sensor-based implant guidance, reinforcing the validity of the present findings within the current body of evidence.

From a clinical diagnostic standpoint, angular deviation thresholds between ±5° and ±7° are frequently cited as practical limits beyond which the risk of biological and prosthetic complications increases [20,21]. Deviations above these values have been associated with a higher likelihood of inferior alveolar nerve injury, sinus perforation, and compromised prosthetic passivity. In the present study, the prototype-assisted condition consistently achieved mean angular deviations below 7° across all planes, whereas freehand placement largely exceeded these thresholds. This situates the proposed system within a clinically acceptable diagnostic safety zone, rather than merely demonstrating statistical improvement.

These findings are in agreement with previous clinical and experimental studies, which have consistently reported that real-time guidance systems contribute to improved implant positioning accuracy and reduced variability, particularly in anatomically complex scenarios [17,19,25].

Unlike optical or robotic navigation systems that rely on expensive infrared cameras or magnetic trackers, the present model integrates compact inertial measurement units (IMUs) with Extended Kalman Filter (EKF) sensor fusion [27]. This configuration provides real-time angular feedback without external calibration or line-of-sight constraints, effectively democratizing digital guidance by reducing both economic and technical barriers. The device thus occupies an intermediate niche between manual and fully digital navigation—retaining manual freedom while offering quantitative guidance.

Complementary Bayesian and equivalence analyses, reported in the Supplementary Materials, further support the statistical robustness and clinical relevance of the observed improvements in angular accuracy.

### 4.2. Mechanisms Underlying Performance Improvement

The improvement in angular precision likely results from two synergistic mechanisms. First, the immediate visual feedback provided by the IMU–EKF system allows real-time microcorrection of drill orientation, reducing cumulative angular drift. This dynamic feedback minimizes proprioceptive uncertainty, analogous to continuous error correction observed in optical navigation [28]. Second, the haptic interface offers tactile and kinesthetic reinforcement, which enhances sensorimotor calibration and consolidates spatial memory—phenomena supported by recent neuroeducational evidence on psychomotor learning [29,30]. These combined sensory channels promote fine motor control and adaptive coordination, explaining the significant effect sizes observed.

These mechanisms are consistent with previous findings in the literature, where real-time feedback and multisensory integration have been shown to enhance motor control, reduce procedural error, and accelerate skill acquisition in simulated and clinical environments [13,31,32].

Additional analyses consistently indicated that the improvement in angular accuracy was primarily driven by the assisted condition rather than by trial order or progressive familiarization, supporting the role of haptic–inertial feedback as the main mechanism underlying procedural refinement. Detailed model outputs are provided in the Supplementary Materials.

### 4.3. Educational and Research Relevance

From an academic standpoint, the integration of inertial feedback into haptic simulation constitutes a pedagogical innovation aligned with competency-based education models recommended by the ADEE [33]. Objective quantification of angular accuracy can transform current dental curricula by enabling continuous assessment of psychomotor performance, reducing instructor subjectivity, and providing real-time benchmarks for skill progression [34,35]. Furthermore, the simulator–prototype system could serve as a standardized research platform to evaluate new implant systems, drilling protocols, or calibration algorithms under reproducible conditions—an aspect often lacking in traditional typodont or cadaveric models [36].

This approach aligns with current trends in competency-based dental education, where objective performance metrics and simulation-based training are increasingly recognized as essential tools for improving clinical skills and standardizing assessment [33,34].

### 4.4. Methodological Considerations and Limitations

While the results are robust, several limitations merit consideration.

First, all procedures were performed by a single experienced operator, precluding assessment of inter-operator variability and learning curves. This design may introduce an operator-related bias, as part of the observed improvement could be influenced by operator expertise rather than solely by the performance of the system. Future trials should incorporate multiple operators with diverse experience levels to examine reproducibility and training transferability [37].

Second, the fixed experimental order (freehand before prototype-assisted) may have introduced a potential learning effect, although mixed-model and sensitivity analyses indicated the device’s effect was independent of trial order. A randomized crossover design would provide stronger internal validity [38]. Therefore, a learning effect cannot be entirely excluded despite the statistical adjustments applied.

Third, the controlled virtual simulation lacks certain clinical perturbations—including limited visibility, soft tissue deflection, bleeding, and electromagnetic interference—that could influence sensor behavior in vivo. In addition, although the simulation environment operates under controlled digital conditions, minor sources of measurement inaccuracy cannot be entirely excluded. These may include system resolution limits, numerical approximations within the simulation software, or minimal sensor latency. However, agreement analysis with the optical reference system demonstrated a very low bias (0.36°) and high precision (RMSE = 0.39°), indicating that any such inaccuracies are negligible and remain well within clinically acceptable limits. Therefore, the findings of this study should be interpreted within the context of a controlled preclinical simulation environment, and their direct extrapolation to real clinical conditions should be approached with caution [39].

In addition, inherent technical limitations associated with IMU-based systems should be considered. Inertial sensors are susceptible to drift over time, which, although mitigated in this study through Extended Kalman Filter (EKF) sensor fusion, cannot be completely eliminated. Accurate performance also depends on proper calibration procedures prior to use, ensuring alignment with the reference coordinate system. Furthermore, environmental factors—such as electromagnetic interference affecting magnetometer readings—may influence measurement accuracy under clinical conditions [40]. While the present controlled simulation minimized these effects and demonstrated high measurement stability, further investigation is required to assess system robustness and long-term stability in real surgical environments.

Finally, although the Kalman filter substantially minimizes drift, IMU sensors inherently exhibit micro-latency (<0.3°) that might affect ultra-precise measurements or prolonged operations [41].

In addition, regulatory considerations must be addressed in subsequent stages. For potential clinical implementation, the device would require ISO 13485 compliance and CE/FDA certification as a Class I medical accessory, ensuring safety and traceability under the Medical Device Regulation (EU 2017/745) [42].

The integration of multivariate (MANOVA), Bayesian, and reliability analyses offers a comprehensive validation strategy. The high reproducibility indices (ICC > 0.95) and strong posterior probabilities of effect (>0.99) provide solid statistical grounds for the generalizability of the results. This reinforces the methodological rigor required for translational applications of haptic–inertial simulators in dental implantology [43].

### 4.5. Clinical Integration and Future Outlook

Beyond its preclinical validation, the present prototype holds significant potential for clinical translation. The system could be integrated as an adjunct to freehand implantology, especially in settings lacking access to full navigation suites. Real-time angular feedback may serve as a safety mechanism to maintain deviation within ±5°, the accepted clinical tolerance that minimizes risk to anatomical structures such as the mandibular canal or maxillary sinus [20,21].

In practical terms, its use could enhance implant parallelism, improve prosthetic passivity, and potentially reduce postoperative complications related to off-axis loading [44]. When paired with digital planning software (CBCT–CAD workflows), the prototype could facilitate semi-guided surgery in both single and multi-implant cases, offering an affordable bridge between analog and fully digital approaches [45].

**Clinical and Technological Translation:**

The proposed system could be seamlessly integrated into existing digital workflows combining CBCT, CAD/CAM planning, and surgical navigation. In clinical environments, the device may complement freehand or semi-guided implant placement, offering real-time angular feedback within the familiar operative workflow [46]. From a technological standpoint, interoperability with digital planning platforms (e.g., DICOM and STL data) would enable precise synchronization between virtual plans and intraoperative execution, paving the way for hybrid, sensor-augmented implantology [47].

From a technological perspective, the system’s data acquisition capabilities open the possibility of machine learning integration for adaptive guidance. Continuous recording of angular trajectories could train predictive models to detect deviations and autonomously suggest corrective cues [47]. Similarly, combining the IMU-based system with augmented reality (AR) or head-mounted displays could create immersive navigation environments that merge real surgical fields with digital overlays, further enhancing precision [48].

**Data Availability and Reproducibility:**

The datasets generated and analyzed during the current study, including calibration protocols and sensor fusion scripts, will be deposited in the Zenodo repository upon acceptance of the manuscript to ensure transparency and reproducibility of the reported findings [48].

## 5. Conclusions and Future Perspectives

This preclinical study confirms that an inertial sensor-based prototype integrated into 3D haptic simulation significantly improves the angular accuracy of dental implant placement, achieving results within the range reported for digital navigation systems while maintaining advantages of portability, simplicity, and low cost. Its dual educational and translational potential positions it as a scalable, evidence-based innovation for both implant training and real-time surgical guidance. By reducing angular deviation to values consistently below commonly accepted clinical risk thresholds, the proposed system provides not only statistical improvement but also diagnostically meaningful guidance with potential implications for anatomical safety and prosthetic predictability.

Future work should focus on clinical validation, regulatory standardization, and software integration toward intelligent, adaptive navigation platforms. Such advancements would contribute to a new generation of smart, sensor-assisted implantology, bridging the gap between manual expertise and digital precision in contemporary oral surgery.

These findings should be interpreted within the context of a controlled preclinical simulation and do not constitute clinical validation.

Further validation through multi-operator studies and clinical trials is required to confirm the system’s effectiveness under real surgical conditions.

## Figures and Tables

**Table 1:** Mixed-effects model summary—Summary of fixed effects and significance for each factor in the model.

Effect	F-Statistic	*p*-Value	Interpretation
Condition (Prototype vs. Freehand)	231.4	<0.001	Highly significant improvement
Plane (Vestibulolingual vs. Mesiodistal)	0.02	0.90	No difference between planes
Condition × Plane interaction	6.1	0.015	Slightly stronger effect in mesiodistal plane

**Table 2:** Bland–Altman agreement and error metrics—Bias, limits of agreement, and absolute error indicators.

Metric	Value	95% Limits or CI	Interpretation
Mean bias (Prototype–Reference)	+0.36°	−0.05° to +0.77°	Minimal systematic bias
SD of differences	0.21°	—	Low dispersion
RMSE	0.39°	—	High precision
MAE	0.31°	—	High accuracy

**Table 3:** Reliability indices—Intraclass correlation coefficients and repeatability parameters.

Reliability Metric	Value	95% CI	Interpretation
Intra-operator ICC (2,1)	0.982	0.965–0.991	Excellent repeatability
Mean absolute difference (°)	0.27 ± 0.19	—	High temporal stability

**Table 4:** Sample size and power analysis—Required and achieved power for clinically relevant differences.

Endpoint	Δ = 2° (80%)	Δ = 2° (90%)	Δ = 3° (80%)	Δ = 3° (90%)
Mesiodistal	94	134	42	60
Vestibulolingual	210	298	93	132
Composite	170	242	80	112

## Data Availability

The raw data supporting the conclusions of this article will be made available by the authors on request.

## Supplementary Materials

The following supporting information can be downloaded at https://www.mdpi.com/article/10.3390/dj14050296/s1, File S1: Section S1 Bayesian Linear Mixed-Effects Model, Section S2 Equivalence Testing (TOST), Section S3 Robustness and Sensitivity Analyses (Bootstrap, Trimmed, Winsorized) and Section S4 Random Forest Predictive Modeling.
