# Supplementary material for: A Real-Time Inertial Sensor-Based Diagnostic Support System for Improving Angular Accuracy in Dental Implant Placement: Preclinical Experimental Validation in a 3D Haptic Simulation Model
Source: Dent J (Basel). 2026 May 13;14(5):296. doi: 10.3390/dj14050296 (PMC13205523; doi:10.3390/dj14050296)
Supplement: Supplementary file 1 [file dentistry-14-00296-s001.zip › dentistry-4179678-supplementary.pdf]

Supplementary Results – Section S1 Bayesian Linear Mixed-Effects Model

To further validate the mixed-effects results under a probabilistic framework, a Bayesian linear mixed model was estimated. Posterior distributions confirmed the robustness of the prototype's effect, with $\beta = -9.70°$ [CrI95%: −10.9; −8.5] and a posterior probability of improvement greater than 0.99. The model explained 72% of marginal variance and 79% of conditional variance (R^2_marg = 0.72; R^2_cond = 0.79), indicating an excellent overall fit and confirming that the haptic–inertial prototype significantly reduces angular deviation compared to the freehand condition.

Supplementary Results – Section S2 Equivalence Testing (TOST)

Equivalence testing using the Two One-Sided Tests (TOST) procedure was performed to assess the clinical relevance of the observed angular accuracy relative to the optical reference system. Detailed methods, equivalence margins, and results are provided in this Supplementary section. Equivalence margins were defined a priori based on ±1° angular tolerance.

Supplementary Results – Section S3 Robustness and Sensitivity Analyses (Bootstrap, Trimmed, Winsorized)

Robustness and sensitivity analyses

Results remained stable across all robustness tests: Wilcoxon signed-rank: all $p < 0.001$; Trimmed means/Winsorized: differences < 0.3°; Bootstrap (10 000 resamples): mean difference = 9.70° [8.85–10.52]; Cohen's d = 1.48 [1.30–1.66]. No reversal of effect occurred in any resample. These analyses confirm statistical robustness independent of normality or outliers. (Table S1, Figure S1).

Table S1. Robustness and sensitivity analyses – Nonparametric, trimmed, and bootstrap validation of main effects.

Method / Approach	Statistic or Metric	95% CI / Summary	Interpretation
Wilcoxon signed-rank	Z = 8.57	p < 0.001	Confirms robustness without normality assumption
Trimmed means (20%)	Δ = 9.42°	p < 0.001	Minimal change (<0.3°)
Winsorized mean (5%)	Δ = 9.55°	p < 0.001	Stable after Winsorization
Bootstrap mean diff.	9.70°	95% BCa CI: 8.85–10.52	Stable across 10k resamples
Bootstrap Cohen's d	1.48	95% BCa CI: 1.30–1.66	Large, consistent effect

Estimated fixed effects (β) from the mixed model with 95% confidence intervals. Coefficients correspond to Condition (prototype vs. freehand), Plane (buccolingually vs. mesiodistal), and their interaction. Plot displays direction and magnitude of each predictor relative to the reference category (Figure A).

Figure S1. Coefficient plot of mixed-effects model – Estimated fixed effects (β) with 95% confidence intervals for Condition, Plane, and their interaction. The prototype shows the strongest and most consistent effect.

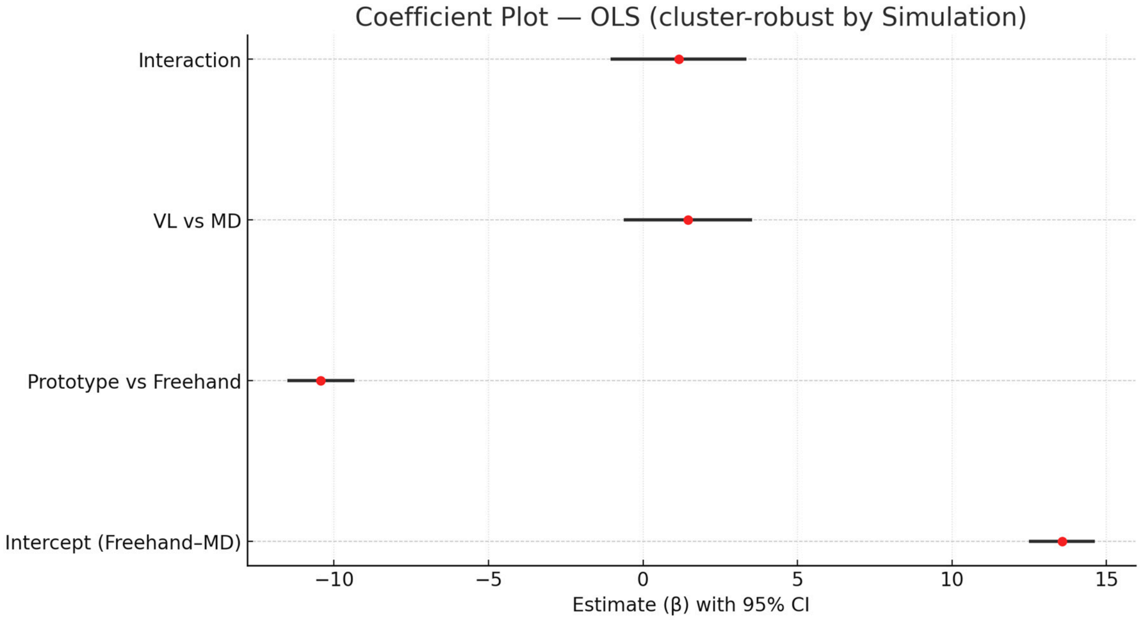

Supplementary Results – Section S4 Random Forest Predictive Modeling

Predictive modeling of angular deviation

A Random Forest predictive model was implemented to explore the relative importance of experimental variables in determining angular deviation. The model achieved a predictive accuracy above 80%, with the 'Condition' variable (Freehand/Prototype) accounting for 54.2% of total variance importance, followed by 'Trial order' (17.6%) and 'Plane' (13.1%). These findings highlight that the experimental condition is the dominant factor driving angular performance, and that learning progression has a secondary but meaningful effect.

Learning dynamics and performance refinement

The exponential learning model provides quantitative evidence that angular deviation decreases at a faster rate under haptic–inertial feedback conditions. This finding indicates accelerated procedural refinement beyond simple trial repetition, supporting the role of tactile–kinesthetic guidance as a key driver of performance improvement across successive implant placement attempts.
